# Cold Gas Spraying of Nickel-Titanium Coatings for Protection Against Cavitation

**DOI:** 10.1007/s11666-020-01139-x

**Published:** 2020-12-23

**Authors:** Georg Mauer, Karl-Heinz Rauwald, Yoo Jung Sohn, Thomas E. Weirich

**Affiliations:** 1 Forschungszentrum Jülich GmbH, Institute of Energy and Climate Research, IEK-1: Materials Synthesis and Processing, 52425 Jülich, Germany; 2 Central Facility for Electron Microscopy (GFE), Institute of Crystallography (IFK), RWTH Aachen University, 52074 Aachen, Germany

**Keywords:** cavitation, cold gas spraying, erosion, nickel-titanium, shape memory alloy

## Abstract

Cavitation erosion is a sever wear mechanism that takes place in hydrodynamic systems. Examples are turbine vanes of hydropower plants or components of valves and pumps in hydraulic systems. Nickel-titanium shape memory alloys (NiTi) are attractive materials for cavitation-resistant coatings because of their pronounced intrinsic damping mitigating cavitation-induced erosion. In this work, NiTi coatings were produced by cold gas spraying. The phase transformation behaviors of the powder feedstock and the as-sprayed coatings were investigated. Regarding the obtained transformation temperatures, the measured substrate temperatures during spraying rule out that either the shape memory effect or the pseudoelasticity of NiTi could affect the deposition efficiency under the applied conditions of cold gas spraying. Another potential effect is stress-induced amorphization which could occur at the particle–substrate interfaces and impair particle bonding by stress relaxation. Moreover, also oxide formation can be significant. Thus, the presence of amorphous phases and oxides in the near-surface zone of particles bounced off after impact was investigated. Oxidation could be confirmed, but no indication of amorphous phase was found. Besides, also the evolution of local microstrains implies that the substrate temperatures affect the deposition efficiency. These temperatures were significantly influenced by the spray gun travel speed.

## Introduction

Cavitation is a mechanism in technical applications that degrades parts in contact with fast-flowing liquids showing rapid changes of pressure (Ref 1). Small vapor-filled cavities can be formed where the pressure is relatively low. If they implode at locations of higher pressure, the fluid has to fill this space again, causing very strong pressure surges, which can be of the order of several 100 MPa. If the steam bubbles collapse nearby or directly on a solid wall, the implosion creates a liquid jet (microjet) that hits the wall at high speed. If this occurs, damage to the component surface can lead to cavitation erosion. Typical examples are turbine vanes of hydropower plants or components of valves and pumps in hydraulic systems. Coating with wear-resistant materials is an effective measure to extend the lifetime of components under cavitation attack.

Nickel-titanium shape memory alloys (NiTi) are attractive materials for applications affected by cavitation erosion because of their pronounced intrinsic damping due to its pseudoelastic performance. Hereinafter, the phase transformation behavior and the corresponding stress–strain performance of NiTi are reviewed to give the necessary background for the discussion of the experimental results.

NiTi is a polymorph-ordered intermetallic compound showing a martensitic-type phase transformation. The high-temperature austenitic phase has a body-centered cubic B2-structure (space group $Pm\bar{3}m$), while the low-temperature martensite has a more complex monoclinic B19’-type structure (space group $P\bar{3}$) (Ref 2). Depending on the thermomechanical history, the trigonal R-phase (space group $P2_{1} /m$) can occur in an intermediate temperature range (Ref 3).

When martensitic NiTi is heated, the change into austenitic phase starts at *A*_*s*_ and is completed at *A*_*f*_ temperatures. Vice versa, when it is cooled, martensite formation starts at *M*_*s*_ and finishes at *M*_*f*_ temperatures. They are different from *A*_*s*_ and *A*_*f*_, respectively. The reversible austenite-martensite transformation takes place in the temperature range from approx. − 50 to 50 °C. The specific transformation temperatures are strongly depending on the Ni-Ti ratio (Ref 4, 5). A variation of the Ni contents by 0.1 at. % changes the transformation temperature by approx. 10 K. Furthermore, the transformation temperatures are very sensitive to impurities (in particular O, C, N).

NiTi alloys exhibit a particular stress–strain behavior unlike conventional metals (Ref 2). Their best known feature is the shape memory effect. If the martensitic phase prevails, i.e., at temperatures close to or below *A*_*s*_, the phase remains martensitic upon deformation. The deformed part does not return to the original macroscopic shape when relieved. Only the small elastic fraction of the strain is reconverted. However, if the alloy is heated after deformation to *A*_*f*_, it transforms to austenite. In this case, it recovers upon subsequent cooling. The part assumes its original macroscopic shape, because the phase changes back to the original twinned martensite (Ref 6).

**Fig. 1:**
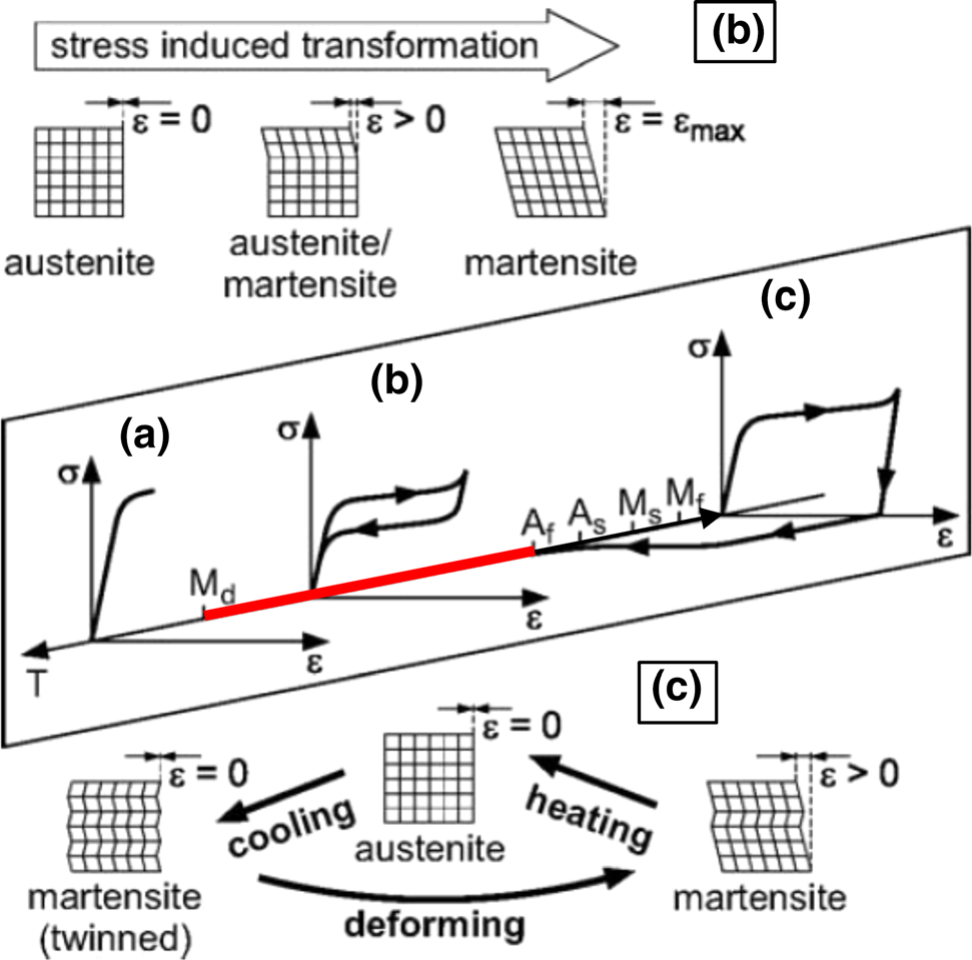
Stress–strain behavior of NiTi alloys depending on the temperature; (a) conventional behavior, (b) pseudoelasticity, (c) shape memory effect (reprinted from (Ref 10), with permission from Elsevier); the bar indicates the temperature range on which pseudoelastic behavior can occur

If the austenitic phase prevails, i.e., at temperatures close to *A*_*f*_ and above, after the first linear deformation the stress remains approximately constant with strain. The deformation is accommodated by the formation of martensite. During subsequent relief, this stress-induced martensite transforms back to austenite. The peculiarity is that the reverse deformation takes place on a distinctly lower stress level. Thus, the stress–strain diagram shows a pronounced hysteresis. Taking into account that NiTi accommodates up to 8% strain (Ref 7), it is obvious that large amounts of elastic energy can be dissipated. The dissipated energy per unit initial volume is given by $E_{\text{dis}} = {\oint }\sigma {\text{d}}\varepsilon$, where *σ* is the engineering stress, *ε* is the engineering strain, and the integration is carried out along the closed loop of loading and relief (Ref 6). This phenomenon of pseudoelasticity (also referred to as superelasticity) is obtained only below a maximum temperature denoted by *M*_*d*_ (martensite desist t., also denoted as martensite deformation t.). Above this critical temperature, the austenitic parent phase is stable enough so that stress-induced martensitic transformation is essentially suppressed (Ref 6). For the investigated NiTi alloy, this temperature was found to be approx. 400 K for quasi-static conditions at a strain rate of 10^−3^ s^−1^ as well as for a strain rate of 1400 s^−1^ which is in agreement with (Ref 8). For another NiTi alloy containing 50.92 at.% Ni, *M*_*d*_ was assumed to be below 373 K (Ref 9). Beyond *M*_*d*_, NiTi performs like an ordinary austenite metal with a relative small elasticity. Figure 1 schematically summarizes the stress–strain behavior of NiTi alloys depending on the temperature.

Besides temperature, also the strain rate significantly affects the pseudoelastic and yielding behavior of NiTi-type shape memory alloys within the pseudoelastic temperature range. Nemat–Nasser et al. have experimentally established the existence of a critical strain rate at which the transition stress for the stress-induced martensite formation equals the yield stress of the austenite phase (Ref 11). For the NiTiCr shape memory alloy investigated in their work, it was found to be in the order of magnitude of 10^4^ s^−1^. At strain rates below this critical level, the material deforms by the formation of stress-induced martensite, accompanied by the yielding of the martensite phase at this critical strain rate, while the material deforms plastically by the dislocation-induced plastic slip at strain rates above (Ref 12).

Okamoto et al. performed in situ studies of crack tips in NiTi tensile specimens (Ref 13). In martensitic as well as in austenitic samples, stress-induced crystalline-to-amorphous transformation was observed at temperatures below 600 K. This upper temperature limit was identified as the transition temperature of ideal glasses. Kinetic glass-transition temperatures of NiTi under rapid quenching conditions are reported in the range of 700-850 K (Ref 14). Huang et al. indicated a glass-transition temperature near 673 K (Ref 15). At higher temperatures, there is a supercooled liquid lacking long-range order in three dimensions as long as no crystallization occurs.

The pronounced pseudoelasticity makes NiTi an important candidate as cavitation-resistant material. Another advantage is the good corrosion resistance. Because of the high costs for manufacturing large NiTi components and their manufacturing challenges, NiTi has been proposed as a coating material. Besides explosive welding, which needs a heat treatment after the process, different attempts were made to manufacture NiTi coatings by thermal spray methods (Ref 2). Atmospheric plasma spraying (APS) was applied to deposit NiTi coatings from mechanical alloyed feedstock powder as reported in (Ref 16). Besides NiTi, they contained considerable amounts of Ni_4_Ti_3_, Ni and some oxides. The cavitation erosion and jet impingement erosion of NiTi coatings manufactured by APS are reported in (Ref 17). Oxides, impurities, cracks and pores in the coatings were found to deteriorate in particular the cavitation erosion protection. The differential scanning calorimetry (DSC) analysis of an as-sprayed coating revealed virtually no phase transformation. These results demonstrate the importance of protective measures against oxidation if NiTi is thermally sprayed under atmospheric conditions. Prealloyed, equiatomic NiTi wires were processed by twin wire arc spraying (TWAS) to manufacture cavitation-resistant coatings (Ref 18). Here, the oxygen uptake could be limited by using argon as shroud gas and for atomization. The best cavitation protection was found for coatings sprayed at shortest distance. Prealloyed NiTi powder was also processed by high-velocity oxy-fuel spraying (HVOF) for mechanical and corrosion protection of structural applications (Ref 19). For the shortest applied spray distance, the highest superficial hardness, the largest adhesion strength and the lowest roughness and porosity were found. The DSC analysis of the sprayed coatings showed a distinct transformation behavior. Thus, the time in-flight of the particles was sufficiently short to limit oxygen pick-up, obviously.

One processing route to coat components with NiTi in inert atmosphere (Ar, 5-7 kPa) is low-pressure plasma spraying (LPPS) (Ref 20), formerly often referred to as vacuum plasma spraying (VPS). In (Ref 21), NiTi coatings were produced by LPPS. A considerably increased cavitation resistance, compared to other established cavitation protection materials, could be demonstrated. However, the characterization of the NiTi coatings exhibited also the presence of small amounts of Ti-rich oxides and intermetallic secondary phases in the coatings. By this, transformation temperatures were slightly shifted since they are very sensitive to variations of the Ni:Ti ratio.

The challenge to limit the oxidation of NiTi suggests to consider cold gas spraying (CGS) (Ref 22) as this is a rather novel thermal spray process with lowest working gas temperatures. Initial CGS experiments using a feedstock mechanically alloyed from elemental Ni and Ti powders revealed that it was not possible to achieve homogeneous and dense coatings (working gas compressed air, 2.7 MPa, 510 °C) (Ref 23). In (Ref 24), the manufacture of NiTi sputter targets by CGS is reported. Again, the feedstock was obtained by mechanical alloying of Ni and Ti. The ball milling process was improved so that only the austenitic NiTi B2-phase could be identified, no elemental Ni or Ti. The working gas was helium (1.5 MPa, 583 °C). The coating showed the same phase composition and similar crystallite sizes and microstrains as found in the feedstock. But the deposition efficiencies achieved were relatively low and the coatings showed cohesion failures as the particles were hardly deformed at impact. In another work, a mechanically blended NiTi powder was cold gas sprayed with compressed air (3 MPa, 500 °C) (Ref 25). Two post-spray treatments, i.e., high-temperature vacuum annealing and friction stir processing, were applied to homogenize the Ni and Ti distributions and to obtain the desired NiTi phase. While porosity formation due to the Kirkendall effect occurred during the heat treatment, the friction stir process produced a defect-free, dense alloyed layer with improved tribological properties. Besides elemental Ni and Ti, the as-sprayed coatings contained intermetallics like NiTi, NiTi_2_ and Ni_3_Ti. The transformation behavior was not investigated.

NiTi can be obtained by thermal spray methods also from elemental Ni and Ti powders by self-propagating high-temperature (combustion) synthesis in which an exothermic, self-sustaining chemical reaction propagates through a premixed powder compact in the form of a high-temperature reaction front (Ref 26). In the past, this was applied to manufacture porous NiTi, e.g., for biomedical applications (Ref 27). In (Ref 28) it is reported that this phenomenon occurred also in cold gas spraying of mechanically alloyed NiTi particles (working gas helium, 2.0-2.5 MPa, 420-580 °C) leading to a partial melting which significantly decreased the deposition efficiency as many particles obviously splashed off the substrate on impact. A significant flashing was observed. The authors proposed to suppress this reaction by lowering the working gas temperature.

The initial state-of-the art as described above was the motivation to further investigate CGS of NiTi, notwithstanding that particular processing difficulties such as poor deposition efficiencies had to be expected. CGS could be an alternative to LPPS if the phase composition and the transformation behavior could be still improved by limiting the oxygen uptake and enhancing the homogeneity of the material.

## Experimental Methods

**Fig. 2:**
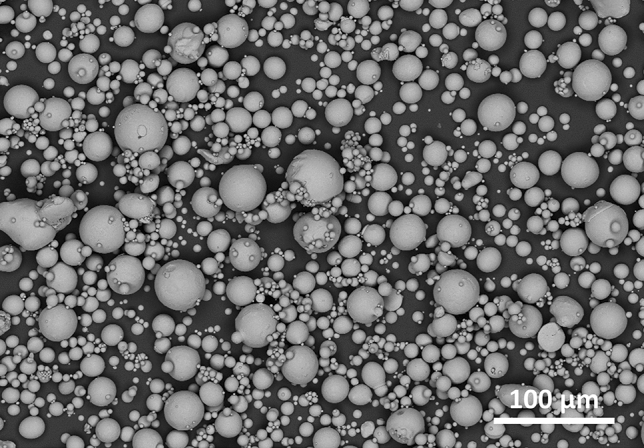
Inert gas-atomized NiTi feedstock powder (backscattered electron image)

The NiTi feedstock powder was inert gas atomized (TLS Technik Spezialpulver, Bitterfeld, Germany) and showed an overall globular morphology, Fig. 2. However, some elongated shapes as well as some satellite particles are obvious. The size fraction − 45 µm was obtained by sieving. The particle size distribution was measured by laser diffractometry (Horiba LA950, Retsch Technology GmbH, Haan, Germany). The characteristic diameters were d_10_ = 10.4 µm, d_50_ = 21.8 µm and d_90_ = 41.9 µm. The oxygen content of 410 ± 0.6 ppm (4 measurements) was determined by infrared (IR) absorption (TCH 600, Leco Corp., St. Joseph, Michigan, USA).

Spray experiments were carried out on an Impact cold spray system 5/11 (Impact Innovations GmbH, Haun/Rattenkirchen, Germany) equipped with a standard D24 de-Laval-type converging-diverging nozzle with an expansion ratio of 5.6 mounted on a six-axis robot. Nitrogen was used as propellant and feedstock carrier gas. The cold spray system allowed a maximum gas temperature of 1100 °C (measured immediately before the gas enters the nozzle, opposite to powder injection) at maximum inlet pressure of 5 MPa. In one experiment a prechamber extension of 170 mm length was tested. Particle temperature estimations performed by the KSS software (Kinetic Spray Solutions GmbH, Buchholz, Germany) had revealed that slightly higher particle temperatures are reached due to the longer dwell time in the prechamber. This effect was more pronounced for the larger particles. After some preliminary tests applying spray distances from the nozzle exit to the substrate surface up to 60 mm, it was set to the minimum of 20 mm. The spray angle was 90°. The gun travel speed for the coating deposition was varied between 125 and 500 mm/s. The raster step size was 1 mm, and the number of deposition passes was varied between 5 and 20. The powder feed rate was set to 26 g/min with a carrier gas flow of 4 m^3^/h. Substrate temperatures were measured during spraying by means of an infrared pyrometer Optris CT laser LT, 8-14 µm (Optris GmbH, Berlin, Germany). The emissivity was estimated as 0.6.

Austenitic-ferritic duplex steel substrates (AISI 318 LN) were grit blasted (alumina, − 600/+ 420 µm) and cleaned in an ultrasonic bath. Deposition efficiencies were determined on the basis of the powder mass feed rate, sample area, gun motion and coating weight considering only the spray time when the gun is over the sample and thus excluding over-spraying. The coating thicknesses were determined by digital image analysis of cross-sectional micrographs using the public ImageJ software (Ref 29).

X-ray diffractometry was carried out by means of the Empyrean system (Malvern PANalytical GmbH, Kassel, Germany). For standard measurements, symmetric theta/theta scans were performed applying the focusing Bragg–Brentano (BB) geometry. Moreover, for grazing incidence measurements (GI), an x-ray parallel beam mirror was attached on the primary optic side, with a fixed divergence slit of 0.25°, and a 0.18° parallel plate collimator was used as a secondary optic in front of the proportional detector. The incidence angle was set to 1°, 5° and 10° to have different information depths. All XRD scans were taken using Cu-K_α_ radiation (45 kV, 40 mA) at Bragg angles 2θ between 25° and 85°, with increments of 0.05° and a scan time of 3 s/step. Thereafter, the data were analyzed using the TOPAS software V4.2 (general profile and structure analysis software for powder diffraction data, Bruker AXS, Karlsruhe, Germany).

DSC analyses were carried out using the DSC 204 F1 Phoenix^®^ system (Netzsch-Gerätebau GmbH, Selb, Germany). According to (Ref 30), one measurement cycle was from room temperature (RT) → + 150 °C (3-min. holding time) → − 150 °C (3-min. holding time) → + 150 °C (3-min. holding time); the heating and cooling rates were 10 K/min.

Scanning electron microscope investigation (SEM) on an Ultra55 model (Carl Zeiss NTS GmbH, Oberkochen, Germany) combined with an energy-dispersive x-ray INCAEnergy355 spectrometer (EDS, Oxford Instruments Ltd., Abingdon, Oxfordshire, UK). For SEM examination, the samples were coated with approximately 2-nm platinum. EDS point analyses were performed with an acceleration voltage of 15 kV.

Sample preparation for in-detail characterization by (scanning) transmission electron microscopy (STEM/TEM) was performed by focused ion beam machining (FIB) using a FEI Strata 400 workstation (FEI Company, Eindhoven, The Netherlands). The TEM investigation of the therefrom received electron transparent FIB cross section was carried out in a ZEISS Libra200FE at 200 kV (Carl Zeiss AG, Oberkochen, Germany) equipped with an in-column Omega filter for electron energy-loss spectroscopy (EELS), an X-Flash detector (Bruker AXS Advanced x-ray Solutions GmbH, Karlsruhe, Germany) for energy-dispersive x-ray spectroscopy (EDXS) and an annular dark-field (ADF) imaging detector (E.A. Fischione Instruments, PA 15632, USA). Image acquisition was done in all operational modes by help of a 2 k × 2 k Ultrascan1000 CCD camera from Gatan (Gatan GmbH, München, Germany).

The cavitation resistance of the NiTi coatings was characterized using an ultrasonic sonotrode in a temperature-controlled bath with distilled water at 22 °C. The sonotrode was operated with a frequency of 20 kHz and an amplitude of 40 µm. The sample surface was placed at a distance of 0.5 mm to the surface of the sonotrode. The test specimen was weighed accurately before testing began and again during periodic interruptions of the test, in order to obtain a history of mass loss versus time (Ref 31).

## Deposition Mechanisms

### Microstructures and Deposition Efficiencies

**Fig. 3:**
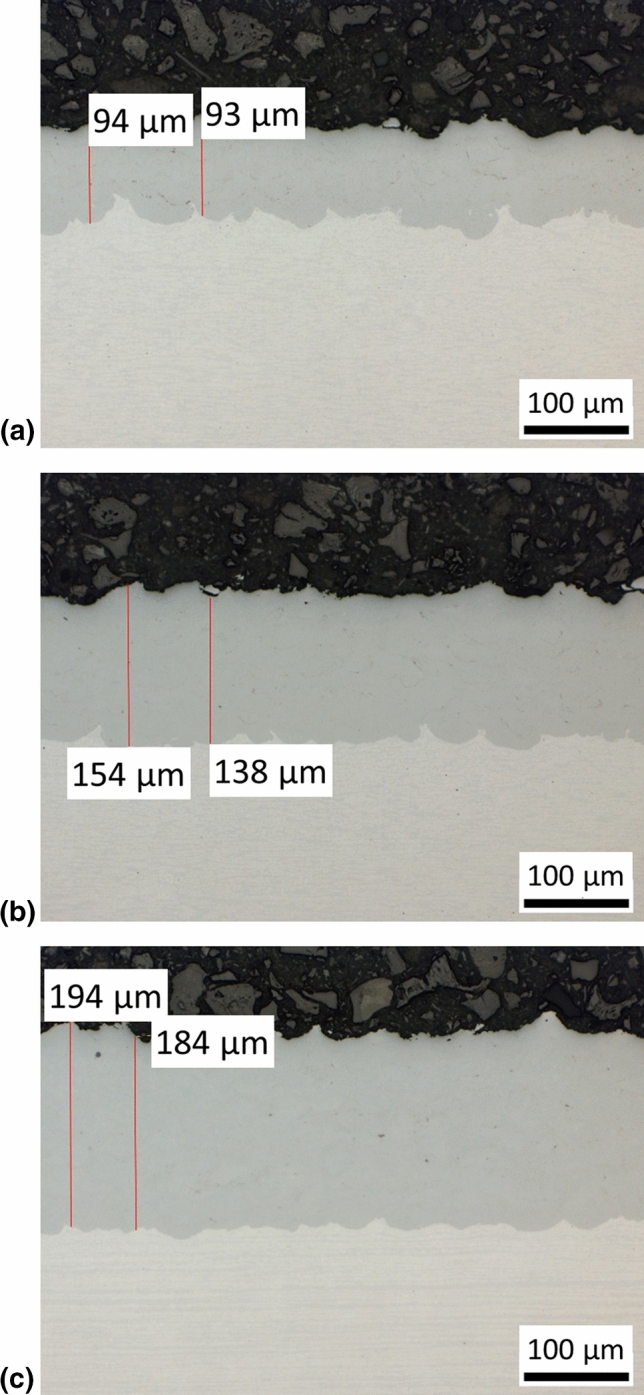
Cross sections of coatings obtained by different combinations of number of spray passes and gun travel speed (optical microscope images); (a) 5 passes with 125 mm/s, (b) 10 passes with 250 mm/s, (c) 20 passes with 500 mm/s

Combining the gun travel speeds of 125, 250 and 500 mm/s with 5, 10 and 20 spray passes, respectively, should have resulted in the same coating thicknesses. But unexpectedly, this was not the case. As the cross-sectional images given in Fig. 3 already show, the coating thickness and thus the deposition efficiency increased with higher travel speeds. In all three cases the coatings were dense and well connected to the substrate.

**Fig. 4:**
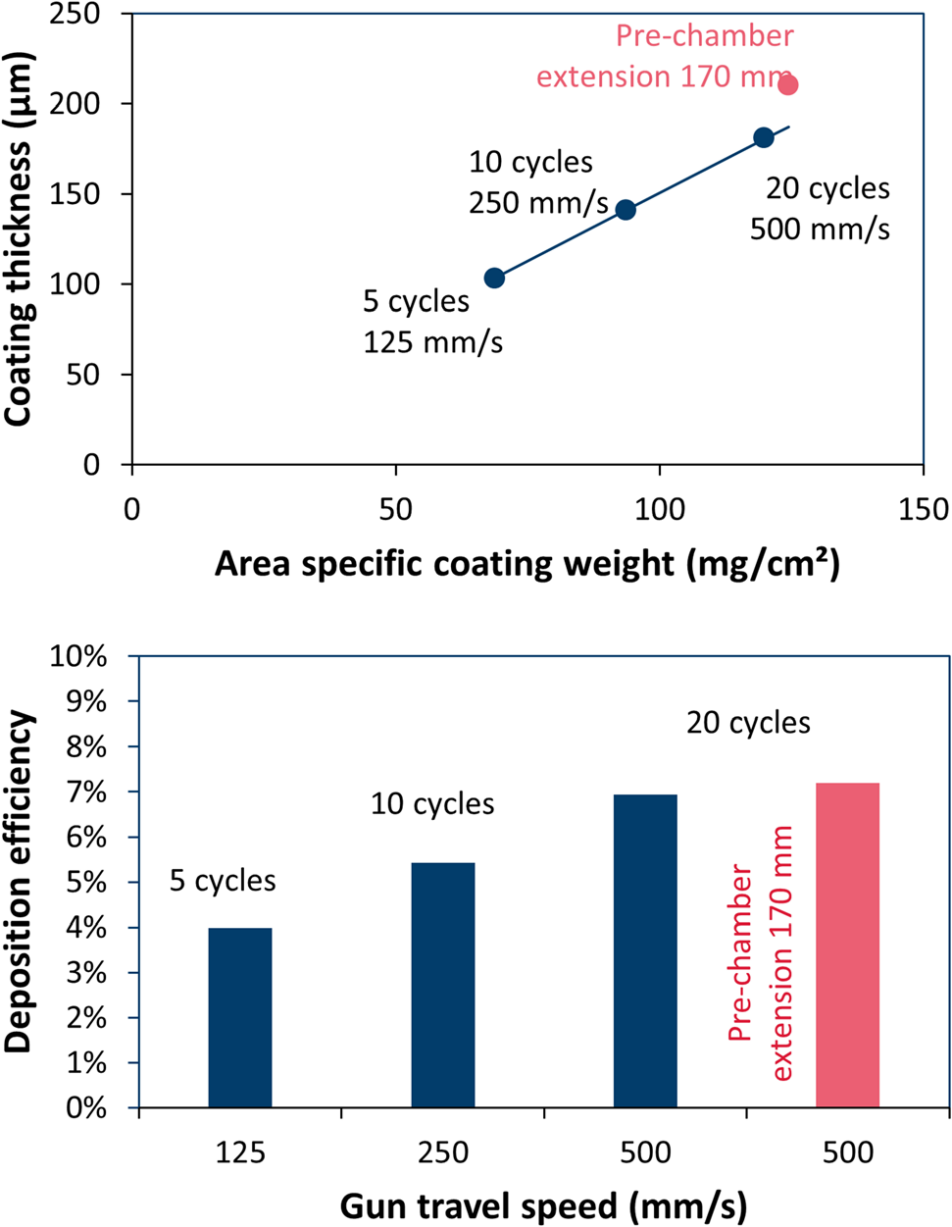
Area-specific coating weights and deposition efficiencies for different combinations of gun travel speeds and number of spray passes; in addition, a prechamber extension of 170 mm was tested with the last parameter set

The quantitative results are given in Fig. 4 and confirm this qualitative finding. The transition from few, thick single layers to several, thin single layers improved the deposition efficiencies significantly. The deposition efficiencies were generally poor. The prechamber extension yielded only a minor benefit.

### Oxidation of Particles and Coatings

**Fig. 5:**
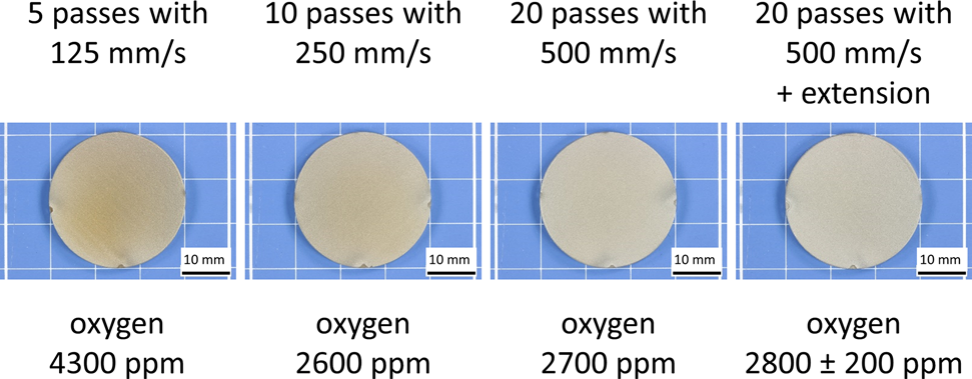
View of as-sprayed samples and oxygen contents determined by IR absorption

The appearance of the as-sprayed samples (cp. Fig. 5) suggests that the substrate temperatures during deposition were different. The coating sprayed with the slowest gun travel speed of 125 mm/s looks darker, indicating more oxidation obviously due to higher substrate temperatures compared to the samples sprayed at higher gun travel speed. The oxygen contents in the coatings determined by IR absorption given in Fig. 5 are in agreement with this trend. However, it must be noted that just one measurement per sample could be made since only very few freestanding coating material was available. Only for the last sample, two measurements were possible. Thus, the results have been rounded to the nearest 100 ppm. The comparison with the oxygen content of the powder (410 ± 0.6 ppm, 4 measurements) proves that the oxygen pick-up was significant.

**Fig. 6:**
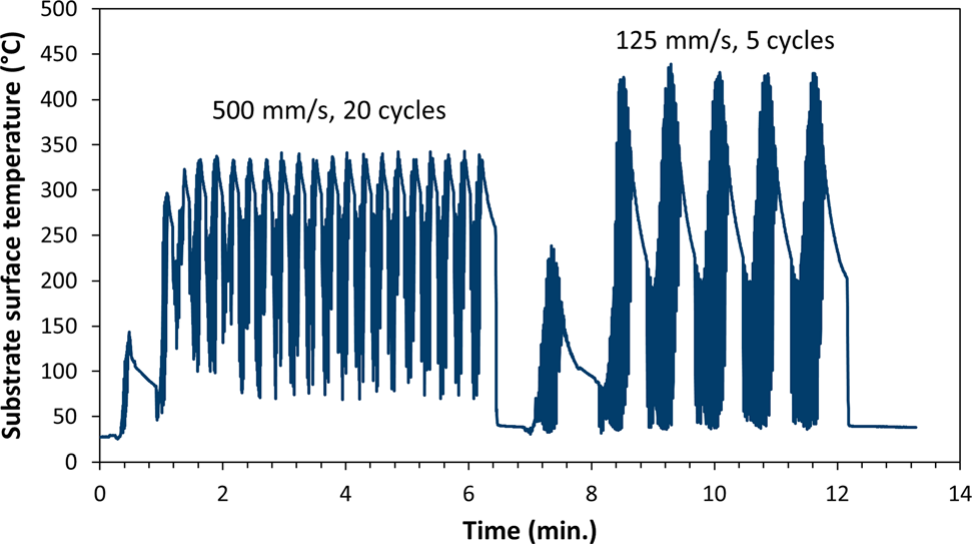
Evolving substrate temperatures during preheating (one cycle) and coating deposition for two combinations of gun travel speeds and number of spray cycles

In fact, pyrometer measurements on the sample surfaces during spraying revealed that the travel speed of the gun had a notable influence on the substrate temperature, Fig. 6. This was in contrast to the spray distance and the gas composition which did not show a significant impact. In case of 500 mm/s, the maximum measured temperature was 343 °C, while for 125 mm/s, it was 439 °C. The dwell time of the particles in the working gas jet and the working gas temperatures were the same for all studied combinations of gun travel speed and number of passes. Therefore, the potential oxygen pick-up during particle’s flight was the same as well. Hence, it can be concluded that the higher oxygen content in the coatings sprayed at higher substrate temperatures was due to superficial oxidation of the coating sublayers immediately after deposition.

**Fig. 7:**
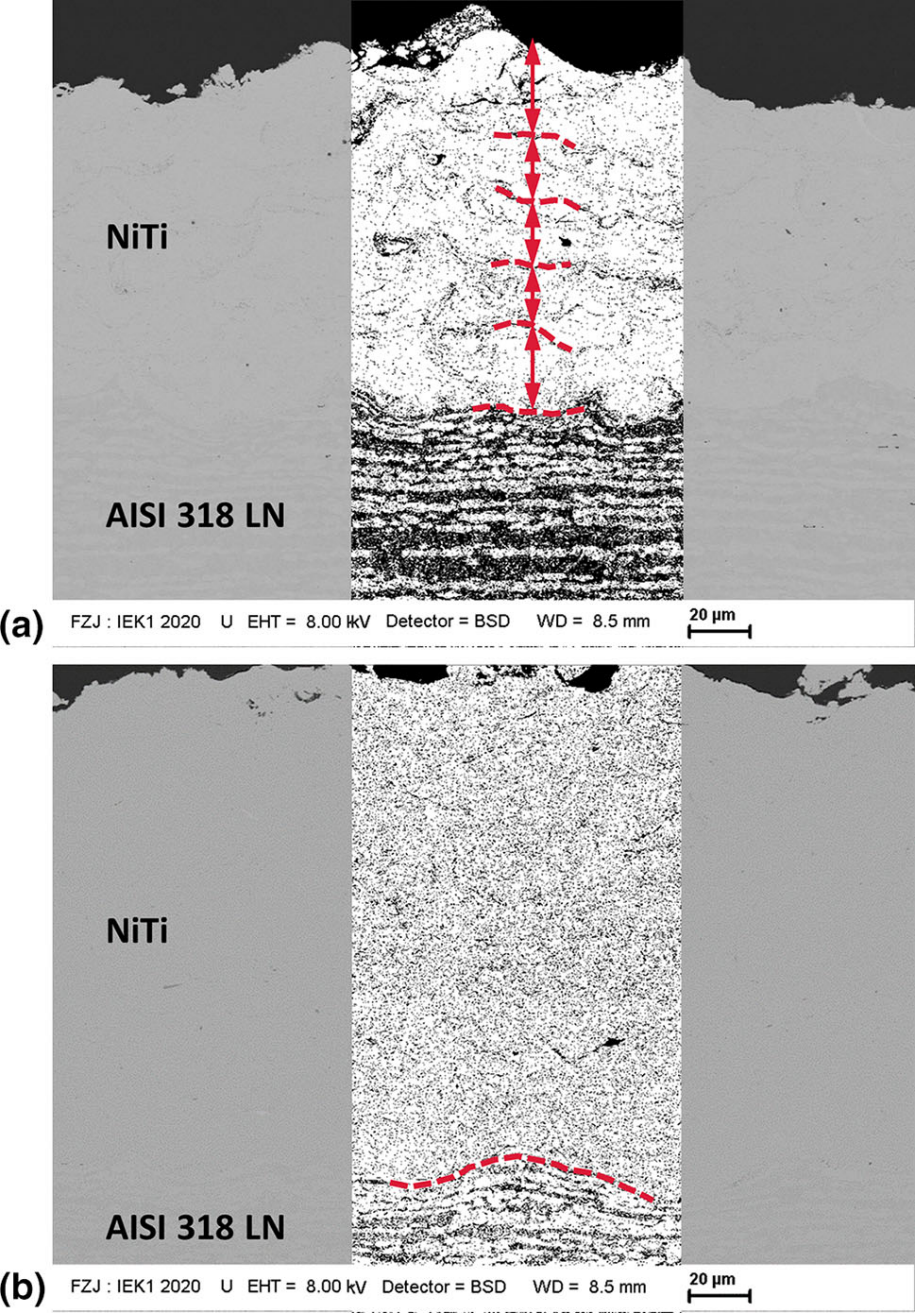
Backscattered electron images of NiTi coatings (a) sprayed in 5 passes with 125 mm/s gun travel speed (5 sublayers are indicated); (b) sprayed in 20 passes with 500 mm/s travel speed (substrate–coating interface is indicated); the middle parts if the images were binarized to enhance the contrast

A closer look into the coatings substantiates the presence of oxides. Figure 7 shows cross sections of two coatings, one sprayed in 5 passes with a gun travel speed of 125 mm/s and the other sprayed in 20 passes with a gun travel speed of 500 mm/s. Due to their lower effective atomic number, oxide appears darker in these backscattered electron images. The first sample shows higher density of oxides at the interfaces between the 5 sublayers and on the coating surface. Such accumulation of oxides at the interfaces is not that obvious in the sample which was sprayed in 20 passes with 500 mm/s. Thus, superficial oxidation of the coating layers immediately after deposition can be assumed for the sample with the higher substrate temperature. Moreover, thinner oxide films were observed in both samples also on the surfaces of the deformed feedstock particles. Apparently, the oxide films on the substrate and particle surfaces are not broken up and removed entirely by the particle impact. Such phenomena of substrate and particle surface oxidation were already reported, e.g., for cold-sprayed alumina particles (Ref 32). It was found that the deformation behavior and consequently the particle bonding were affected.

**Fig. 8:**
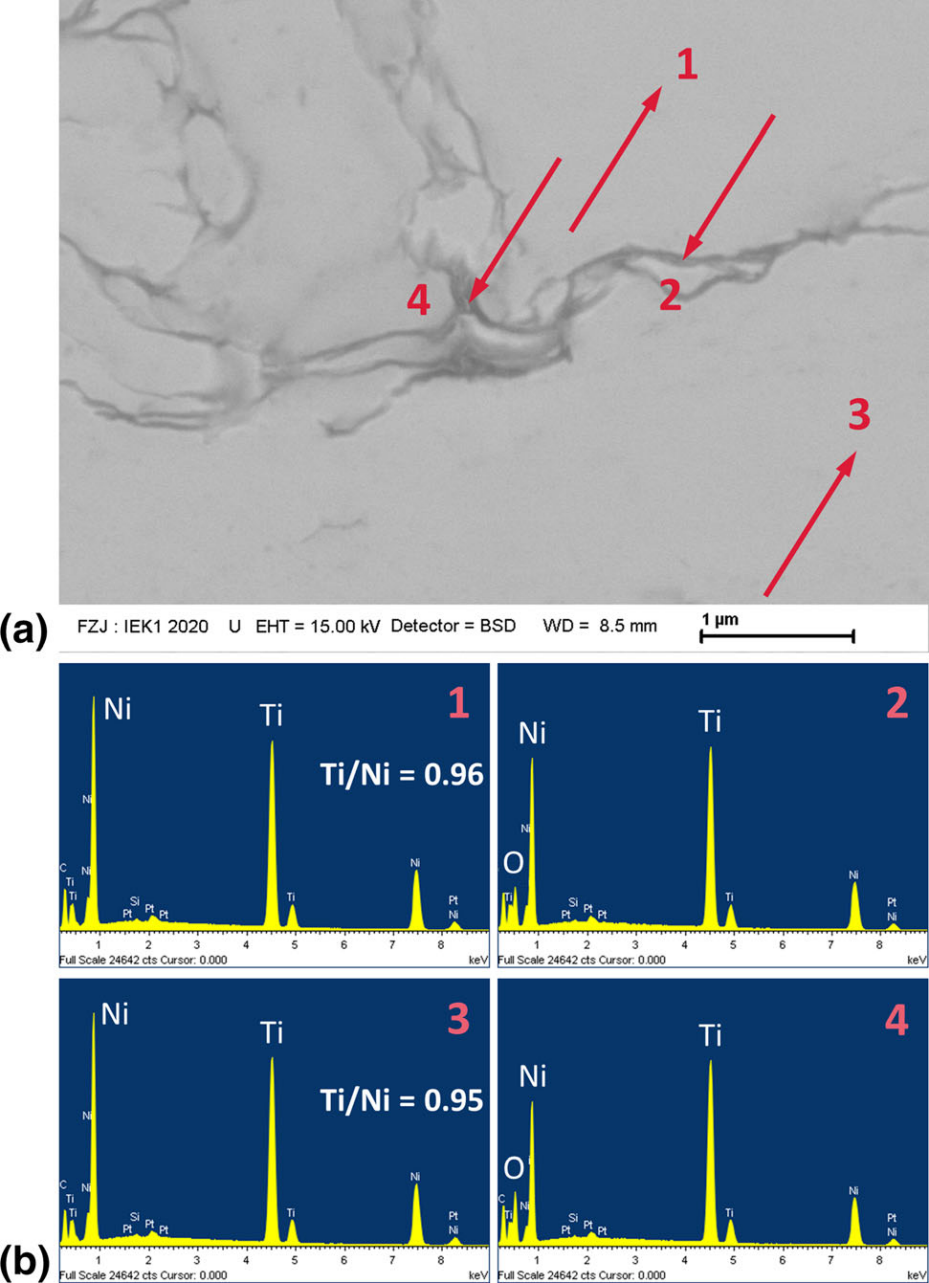
EDS point analyses of a NiTi coating sprayed in 5 passes with 125 mm/s gun travel speed; (a) backscattered electron image with EDS positions 1-4 indicated by arrows; (b) energy spectra 1-4 with highlighted Ni, Ti and O peaks; the quantitative Ti/Ni atomic ratios could not be given reliably for the oxide films (spectra 2 and 4) due to their small thicknesses compared to the dimensions of the material volume excited by the electron beam

Figure 8 gives a cross-sectional backscattered electron close-up and some EDS point analyses of the sample sprayed in 5 passes with 125 mm/s gun travel speed. The non-oxidized fractions show a Ti-Ni atomic ratio similar to the one in the feedstock powder. In contrast, the EDS analyses of the oxide films reveal that their composition is shifted to higher Ti:Ni atomic ratios. Additionally, an oxygen peak is evident. This suggests the formation of Ti-rich oxides like Ni_2_Ti_4_O (Ref 20).

**Fig. 9:**
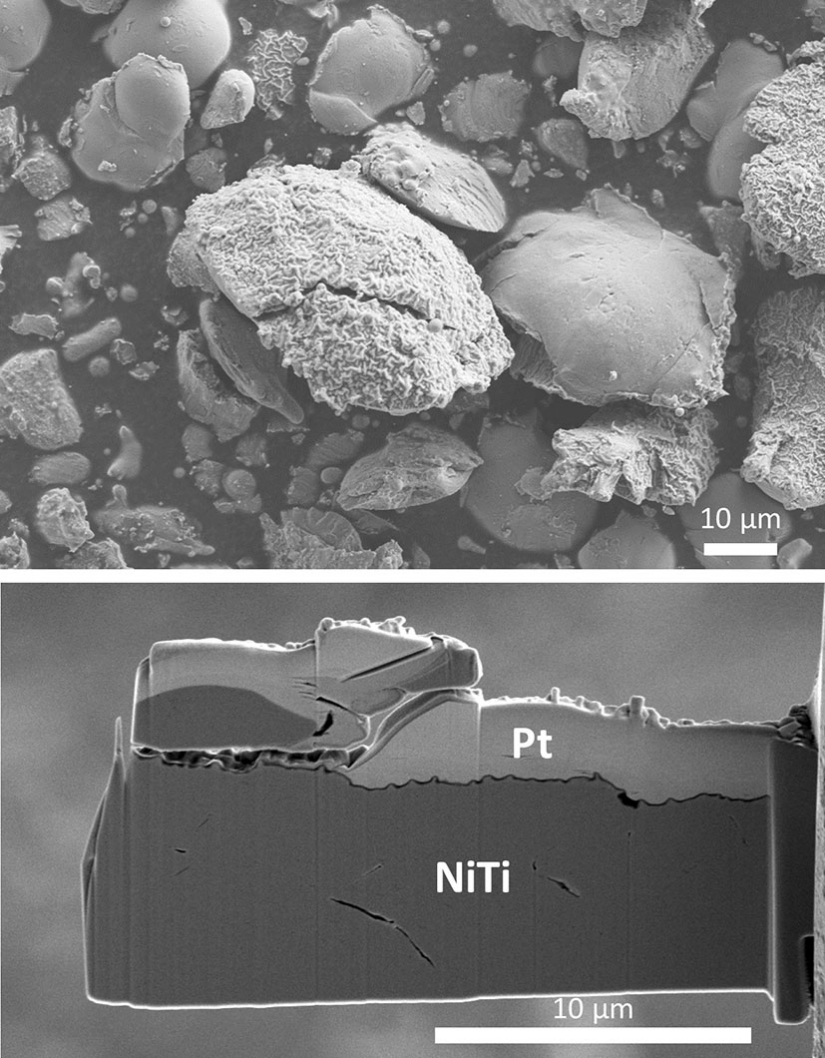
Non-bonded particles bounced off after impact on the substrate, collected during spraying on adhesive carbon pads (secondary electron image, top); FIB-sectioned lamella representing a cross section through a typical rippled particle surface (bottom)

To have a closer look at the phenomena on the particle–substrate interface, non-bonded particles bounced off after impact on the substrate were collected during spraying on adhesive carbon pads in front of the sample stage. Figure 9 (top) shows that they are lens-shaped flattened. Most particles show a smooth surface on the one side and a rippled surface oppositely. The latter is assumed to have been formed at particle impact on the substrate face.

**Fig. 10:**
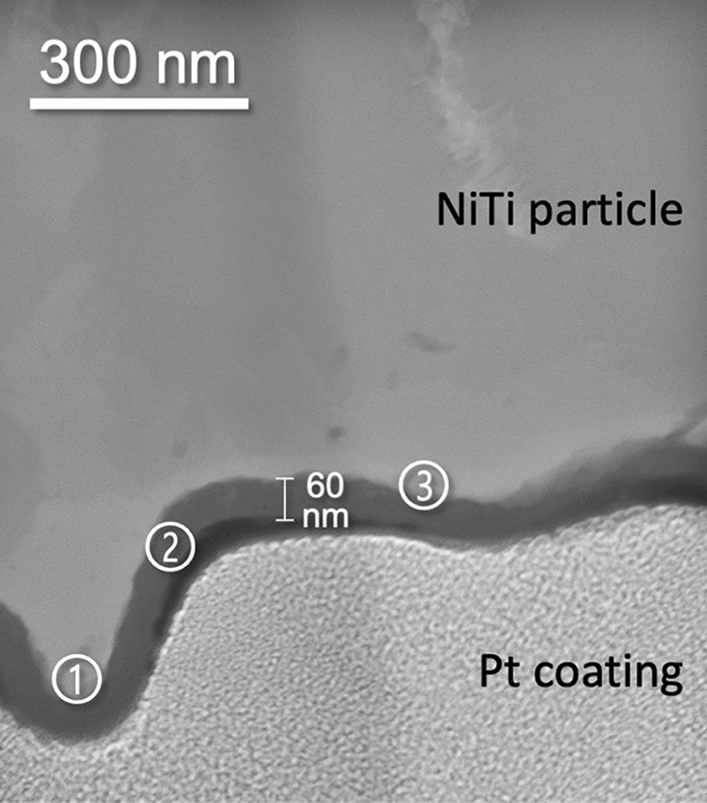
STEM high-angle annular dark-field (HAADF) image of the rippled surface region of a typical NiTi particle; the circled numbers indicate the positions for the performed EELS analysis (Fig. 10)

A TEM cross-sectional sample from the rippled surface of a typical NiTi particle was prepared by FIB (Fig. 9, bottom) and subsequently investigated by scanning transmission electron microscopy (STEM). Figure 10 shows the STEM high-angle annular dark-field (HAADF) image of the rippled surface region. Since the local intensity of HAADF images is roughly proportional to the square of the mean atomic number, the meandering band of lower contrast indicates that the NiTi particle is covered with a continuous surface layer of about 60 nm in thickness. Hence, selected area electron diffraction patterns and EEL spectra were recorded for further identifying the nature of this surface layer.

**Fig. 11:**
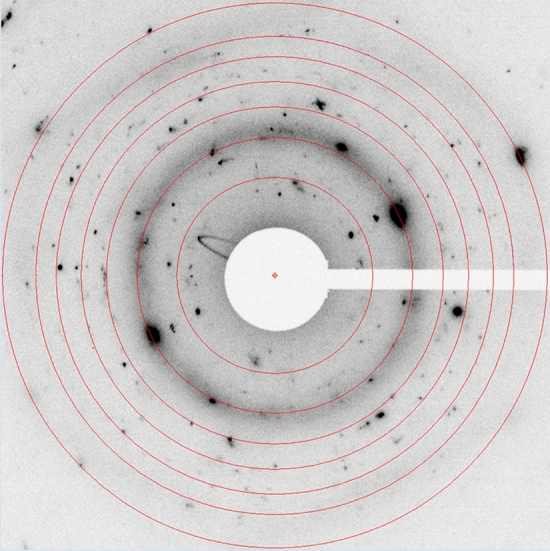
Selected area electron diffraction (SAED) pattern recorded from the detected surface layer in Fig. 10; the overlaid red rings mark the positions of the expected diffraction spots of NiTi with B2 (CsCl-type) structure [PDF 01-076-3614 (Ref 33)]

The evaluation of the selected area electron diffraction (SAED) pattern (Fig. 11) recorded from the rim area in Fig. 10 shows aside reflections of NiTi with B2 (CsCl-type) structure numerous small diffraction spots which indicate the formation of at least one secondary phase with small grain size in the nanometer range. However, it was not possible to find a unique and good match of the corresponding set of extracted interplanar d-spacings with one of the phases listed in the present version of the PDF-4 + 2020 database (International Centre for Diffraction Data (ICDD), Newtown Square, PA, USA) (Ref 33). Nevertheless, since no other heavy element aside nickel and titanium could be detected by EDXS at the surface-near region and the diffraction data showed some resemblance, the formation of nickel-titanium oxides such as Ti_4_Ni_2_O_0.3_ [PDF 00-055-0272 (Ref 33)] or NiTiO_3_ [PDF 00-033-0960 (Ref 33)] is suggested. This leads to the conclusion that the visible surface layer is result of partial oxidation of the NiTi particle.

**Fig. 12:**
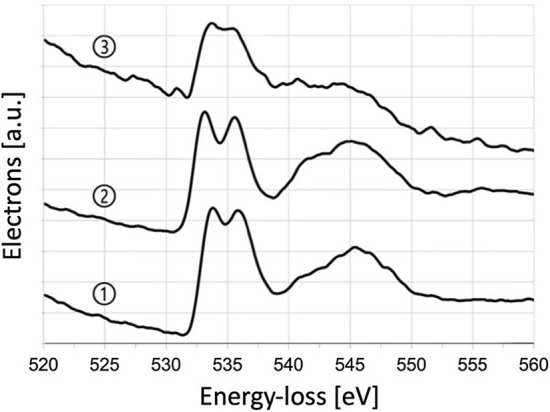
EEL spectra (recorded in STEM mode) from the surface layer showing the region of the O-*K* edge at energy loss of about 532 eV. The circled numbers refer to the positions of the EELS analysis indicated in Fig. 10

This hypothesis was checked by recording EEL spectra at three positions at and close to the detected surface layer (Fig. 10). All three EEL spectra clearly prove presence of oxygen in the surface layer and surface-near region as shown in Fig. 12. This supports the above-made assumption. The pronounced fine structure of the O-*K* ionization edge indicates a rather high crystallinity of the formed nickel-titanium oxides, which is in agreement with the presence of sharp tiny diffraction spots of the secondary phases (see Fig. 11). It should be noted that a corresponding check by EELS for a possible formation of nitrogen containing phases was negative in all cases.

Although it cannot be ruled out that oxidation of feedstock particles could also occur after bouncing off, it is assumed that the oxide films on the surfaces are formed predominantly in-flight as the temperatures are higher than after impact. Together with the oxides on the substrate surface, they could contribute to the poor deposition efficiencies generally found at cold gas spraying of NiTi. From the investigation of the rebounded particles, it is confirmed that the oxide films are not completely broken up and removed at impact.

### Effect of Possible Phase Transformations in Impacting Particles

**Fig. 13:**
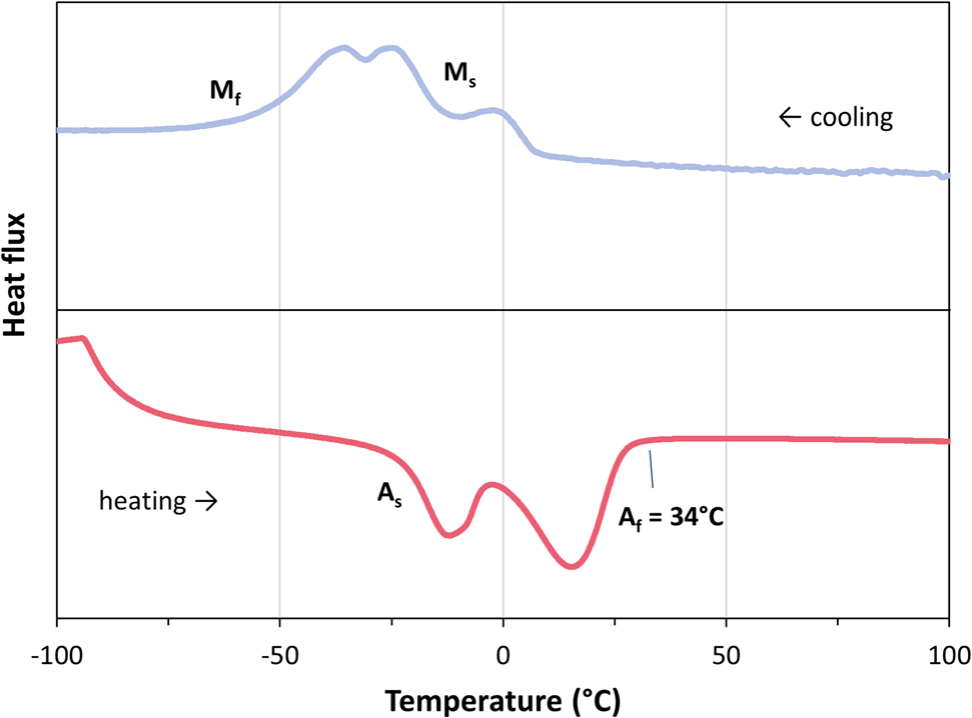
DSC analysis of the NiTi feedstock powder

**Fig. 14:**
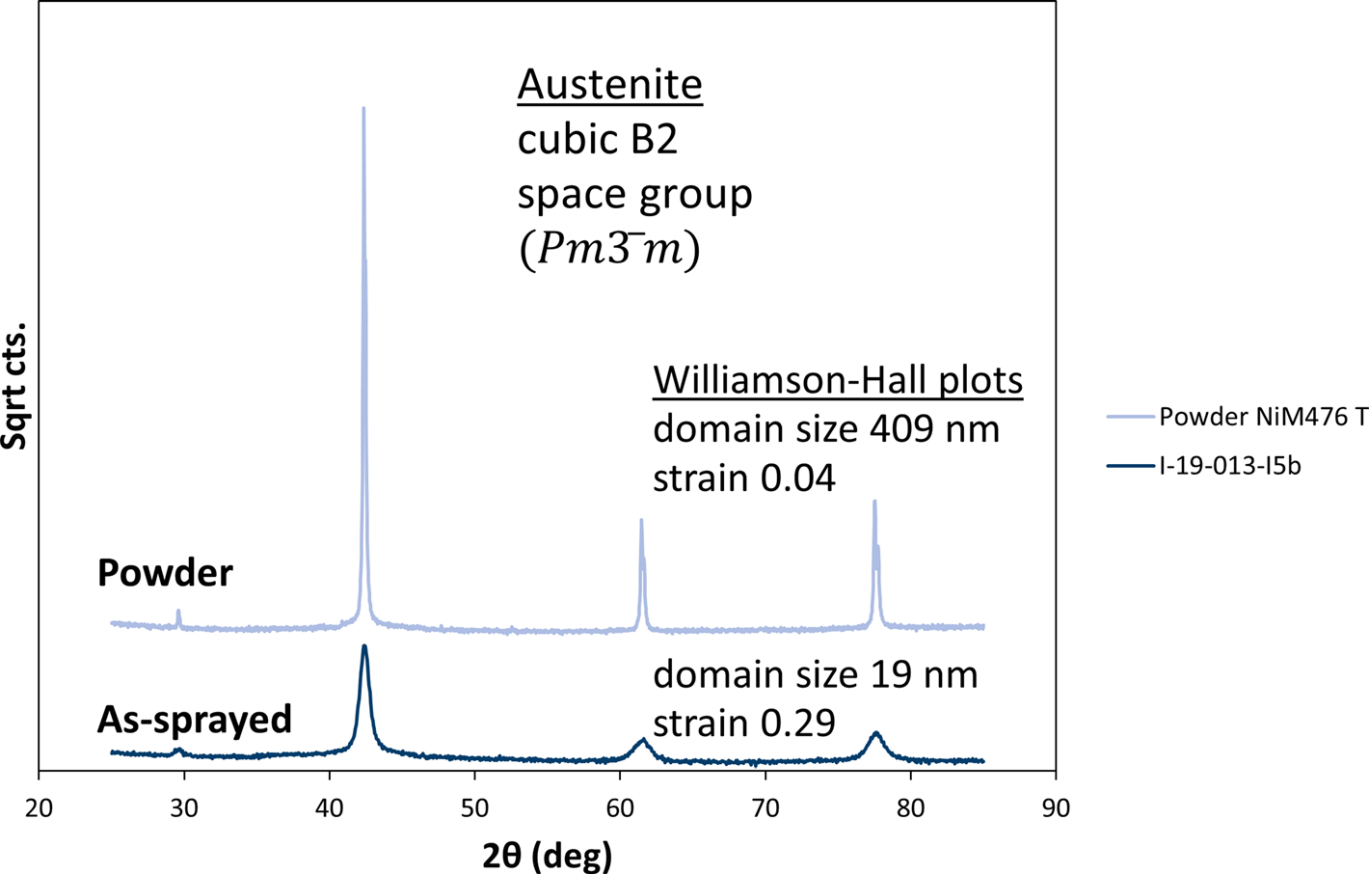
XRD results of the NiTi feedstock powder and one of the as-sprayed NiTi coatings (20 passes with 500 mm/s, with prechamber extension)

Figure 13 gives the phase transformation behavior of the NiTi feedstock powder obtained from differential scanning calorimetry (DSC). Definitely, the particle temperatures during heating by the working gas are above *A*_*f*_ ≈ 34 °C. Thus, the impacting particles can be considered as austenitic. This was confirmed by the results of the x-ray diffraction analysis (XRD) given in Fig. 14. Hence, the feedstock is single-phase austenitic already before heating in the gun. This means that the shape memory effect cannot be effective in the impacting particles since it is initiated in the martensitic phase (cp. "Introduction" section). This is true for all the three investigated parameters as the particle temperatures (and velocities) upon impact were the same. Also the collected off-bounced particles were virtually single-phase austenitic (XRD results are not shown here). Obviously, phase transformations in the impacting feedstock particles did not take place and thus did not impair the deposition efficiencies.

### Effect of Possible Phase Transformations in Deposited Layers

**Fig. 15:**
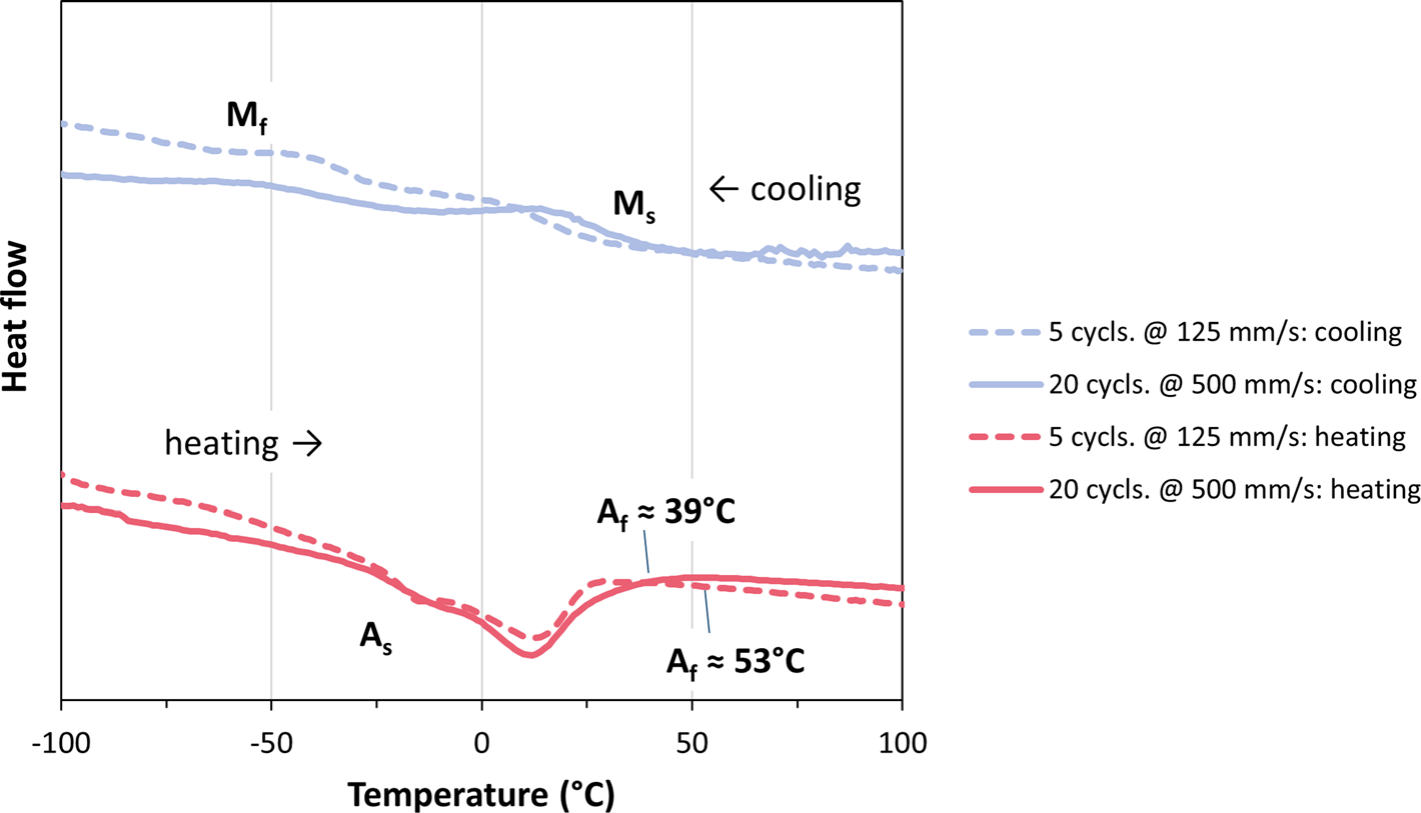
DSC analysis of two as-sprayed NiTi coatings

Besides possible phase transformations in impacting particles, the deposition efficiencies could be affected also by phase transformations and the occurrence of pseudoelasticity or the shape memory effect in the deposited layer on the substrate. The XRD analysis (Fig. 14) of the phase composition in the as-sprayed coating revealed that it was purely austenitic. Another result is that in the as-sprayed coating, the crystallite domain sizes were quite small and there was some strain due to residual stresses. In Fig. 15, the DSC analyses of two as-sprayed NiTi coatings are given. The transformation temperatures could be clearly identified. Compared with the powder (see Fig. 13), the transformation peaks are slightly shifted upwards. This is suggested to be an effect of the much smaller crystallite domain size in the as-sprayed coatings than in the powder (cp. XRD results given in Fig. 14) as the particles were highly deformed at impact. The kinetics of phase transformations might be impeded due to the dense network of grain boundaries. Furthermore, as the strains are likely to vary locally, the DSC peaks were less pronounced and broadened. Annealing probably would lead to homogenization and thus sharpen the DCS peaks and smoothen the plots as shown in (Ref 21).

Although the transformation peaks are slightly shifted to higher temperatures, it is definite that only temperatures above *A*_*f*_ occur in the substrate, while new layers are deposited on top of the coating, cp. "Oxidation of Particles and Coatings" section. After deposition, the samples cooled down to room temperature, but the range of martensite formation was not reached, obviously. Hence, after spraying the first layer, the deposition took place on austenitic NiTi. From this, it can be ruled out that the shape memory effect is active in the previously deposited NiTi layers, as assumed already for the particles (cp. "Effect of Possible Phase Transformations in Impacting Particles" section).

The measured temperatures were generally above *M*_*d*_ (cp. "Introduction" section). Under such conditions, also pseudoelastic effects cannot take place and cause the dissipation of a considerable portion of the particles’ kinetic energy. For the same reason, the use of a heated sample stage in some preliminary tests (substrate temperature was increased up to the oxidation limit of NiTi at approx. 400°C) did not have a positive effect on the deposition efficiency (results are not shown here).

### Possible Stress-Induced Formation of Amorphous Phases

The stress-induced formation of amorphous phase may be possible close to the particle-substrate interface where shear instabilities occur at impact. Koike et al. explicitly suggest that an amorphous state can be caused in NiTi by the shear instability associated with a high dislocation density affecting the crystalline stability (Ref 34, 35). Typical temperatures in these shear bands at particle impact obtained by modeling (Ref 36) can exceed the kinetic glass-transition temperature (cp. "Introduction" section) feasibly. As a result, thin layers of supercooled viscous material might emerge which could impair particle bonding and affect the deposition efficiency, because near the glass-transition temperature, an atomic-level rearrangement occurs referred to as structural relaxation (Ref 15). Slower travel speeds and thus higher substrate temperatures could promote this effect which would be in agreement with the experimental results.

Therefore, indications for the presence of amorphous phase were searched for close to the rippled surface of the off-bounced particle. However, the (SAED) pattern (Fig. 11) recorded from the rim area did not prove such suggestion.

### Micro- and Macrostrains in As-Sprayed Coatings

Residual strains in the as-sprayed coatings were analyzed by XRD using the common focused Bragg–Brentano (BB) configuration as well as three grazing incidence (GI) parallel beam setups with entry angles of 1°, 5° and 10°. While the penetration depth of the x-rays is several tens of micrometers for the BB configuration, it was estimated as 0.7, 3.4 and 6.8 µm, respectively, for the three GI angles. In contrast to macrostrains (uniform) which are obtained by analysis of the peak positions, the microstrains (non-uniform) effect like local defects in the lattice.

**Fig. 16:**
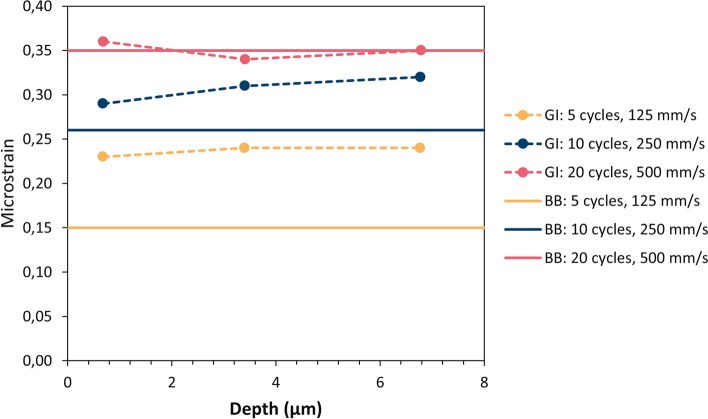
Residual microstrains (non-uniform) in the as-sprayed coatings; GI: grazing incidence (dashed lines), BB: Bragg–Brentano configuration (solid lines)

Figure 16 shows the microstrains (non-uniform) which were obtained by peak broadening analysis of the x-ray diffractograms. As already mentioned, bonding in cold-sprayed coatings occurs due to large strains which are present close to the particle–substrate interfaces, but not in the core regions of the deposited particles. Thus, the level of non-uniform microstrains can be an indication for the intensity of particle bonding and thus the deposition efficiency. In fact, the largest microstrains were found in the sample with the best deposition efficiency, which was sprayed with the highest gun travel speed at the lowest substrate temperatures. For this sample, there was virtually no difference between the GI and the BB results, i.e., there was no strain gradient by depth. Lowering the travel speed and the number of spray passes resulted in smaller microstrains as well as in the development of strain gradients. It is assumed that this was due to temperature-induced relaxation of the microstrains, which was more pronounced in deeper layers since they were heated more often during spraying than the layers near the surface which were deposited at last. These results confirm that the substrate temperature plays an important role for the deposition efficiency not only due to the formation of oxides but also with respect to the microstrains in the near-interface region.

**Fig. 17:**
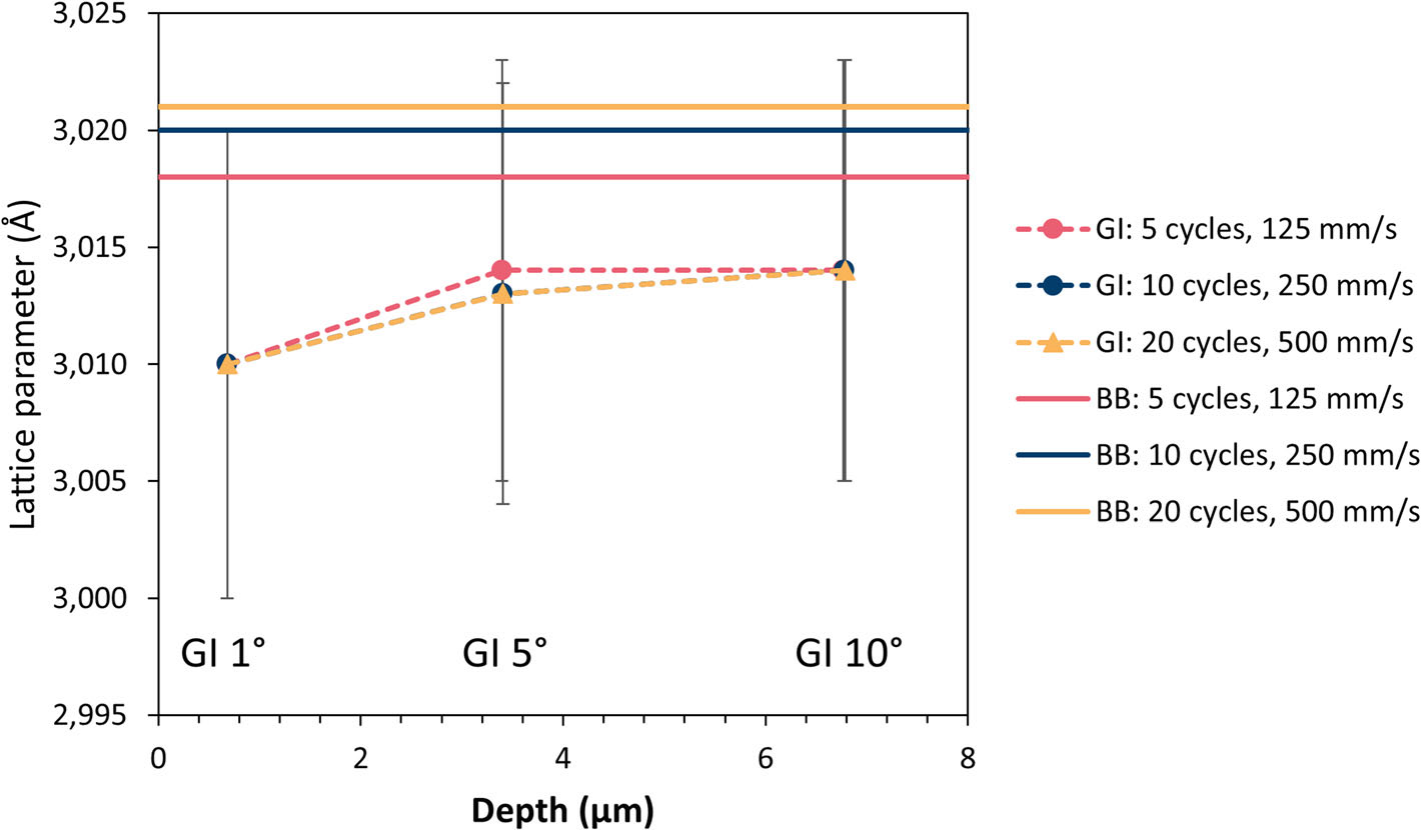
Lattice parameters (uniform) in the as-sprayed coatings; GI: grazing incidence (dashed lines), BB: Bragg–Brentano configuration (solid lines)

Unlike microstrains, uniformly distributed residual macrostrains affect the peak positions in the x-ray diffractograms and thus are represented by the lattice parameters. In Fig. 17, they are given for the same three samples dealt with above. There was virtually no influence of the gun travel speed and number of spray passes. Obviously, the substrate temperatures did not influence the residual macrostrains. Thus, there is no interdependency between macrostrains and deposition efficiency. In all three samples, there is a gradient in the direction of coating thickness. In larger depth (BB results), the macrostrains were higher than close to the surface since they were accumulated layer by layer during spraying.

## Cavitation Tests

**Fig. 18:**
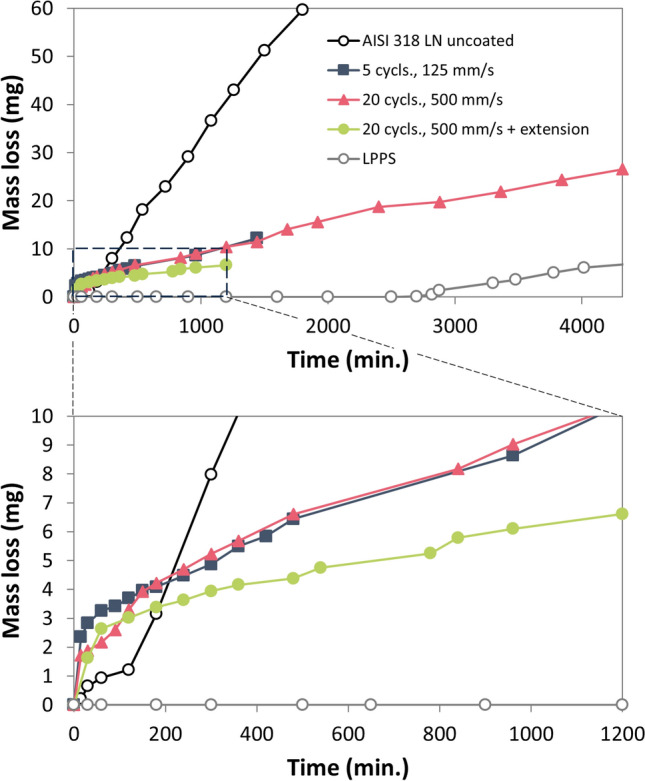
Cavitation-induced mass losses of the uncoated substrate material AISI 318 LN, three coatings manufactured by CGS (this work) and a LPPS sample (Ref 21): complete chart (top) and figure detail (bottom)

Figure 18 gives the results of some initial cavitation tests according to (Ref 31) as described in "Experimental Methods" section. The cavitation-induced mass losses of the uncoated substrate material AISI 318 LN, three coatings manufactured by CGS (this work) and a LPPS sample (Ref 21) are plotted versus time. An interesting detail is that the CGS coatings initially exhibited a higher erosion rate than the uncoated material. The early removal of some weakly bonded particles at the coating surface which were not peened in successive coating cycles may be the reason. Subsequently, the erosion rate stabilized at a much lower level than the uncoated sample. In the end, the bare steel was outperformed by far. The two samples sprayed with 5 cycles at 125 mm/s gun travel speed and 20 cycles at 500 mm/s gun travel speed showed almost the same behavior. The third sample sprayed with 20 cycles and 500 mm/s gun travel speed using the prechamber extension showed the best results among the CGS samples. For these conditions, also the largest deposition efficiency was obtained (Fig. 4).

The excellent performance of LPPS samples has not yet been achieved. With LPPS, a measurable loss of mass could only be detected after 2700 min. After that, however, this sample showed a similar erosion rate to the CGS sample, which was sprayed with 20 cycles at 500 mm/s. One reason for the immediate onset of erosion on the CGS samples could be the limited strength and elongation at break of the cold-sprayed structures due to imperfect cohesion at the south poles of the particles. This is known from the additive production by CGS (Ref 34). The situation can be improved by post-treatment (hot isostatic pressing, annealing) (Ref 37).

## Conclusions and Outlook

CGS was tried as an alternative manufacturing process for cavitation-resistant NiTi coatings. The coatings were dense and well connected to the substrates. However, the observed deposition efficiencies were generally poor (below 10%). Based on the investigated phase transformation temperatures, the shape memory effect and pseudoelasticity in the particles and in the near-surface zone of the substrates could be ruled out as possible reasons for this. Another possibility is that the material could get locally amorphous due to the shear instabilities occurring at the particle–substrate interfaces upon particle impact. However, no indication for the presence of amorphous phase could be found in the near-surface zone of off-bounced particles.

The spray gun travel speed had a considerable impact: the change from few, thick single layers to several, thin single layers improved the deposition efficiencies significantly. This was assumed to be due to the lower measured substrate temperatures and the reduction of the oxygen uptake at the sample face immediately after deposition. This may have improved particle bonding. Vice versa, lowering the travel speed and the number of spray passes led to higher substrate temperatures and more surface oxidation. Moreover, it resulted in smaller local microstrains as well as in the development of strain gradients. It is assumed that this was due to temperature-induced relaxation which might impair particle bonding and thus the deposition efficiency. Active substrate cooling could be an option to improve this situation.

Although these results shed light on some mechanisms (and rule out others), the generally low level of the deposition efficiencies obtained with NiTi in CGS could not be substantiated only by the surface oxidation of particles and substrates and temperature-induced relaxation. In addition, the mechanical strength of NiTi in dependence of the particle size can be another explanatory approach. It can be investigated in a special compression test using a modified nanoindenter (Ref 38). Furthermore, the very high strain rates in CGS are another issue with unknown effects on the deposition efficiency so far.

Initial cavitation tests proved the potential of cold-sprayed NiTi as cavitation-resistant coating material. The uncoated reference sample of the substrate material (AISI 318 LN) was outperformed by far. However, the excellent performance of the LPPS samples obtained in (Ref 21) has not yet been achieved. This is because the onset of cavitation erosion occurred in the CGS samples immediately after the start of the tests, whereas it was considerably retarded in the LPPS sample. Upon erosion onset, CGS and LPPS samples performed at similar wear rates.
